# Tissue factor, factor VIII and IX in microvesicle-induced thrombosis and tumor growth of pancreatic cancer

**DOI:** 10.1186/s12959-025-00715-x

**Published:** 2025-04-11

**Authors:** Sheng-Chieh Chou, Shu-Lun Chang, Cheng-Yeh Yu, Chao-I Lin, Yen-Ting Chang, Li-Fu Chen, Jia-Yi Li, Chen‑Hsueh Pai, Shu-Rung Lin, Wern-Cherng Cheng, Chang-Tsu Yuan, Shu-Wha Lin

**Affiliations:** 1 Division of Hematology, Department of Internal Medicine, National Taiwan University Hospital, Taipei, Taiwan; 2 Graduate Institute of Clinical Medicine, College of Medicine, National Taiwan University, Taipei, Taiwan; 3 Department of Clinical Laboratory Sciences and Medical Biotechnology, National Taiwan University, Taipei, Taiwan; 4 Department of Bioscience Technology, College of Science, Chung-Yuan Christian University, Taoyuan, Taiwan; 5 Center for Nanotechnology, Chung-Yuan Christian University, Taoyuan, Taiwan; 6 Department of Laboratory Medicine, College of Medicine, National Taiwan University Hospital, National Taiwan University, Taipei, Taiwan; 7 Department of Laboratory Medicine, Mackay Memorial Hospital, Taipei, Taiwan; 8 Department of Pathology, National Taiwan University Cancer Center, Taipei, Taiwan; 9 Department of Pathology, National Taiwan University Hospital, Taipei, Taiwan

## Abstract

**Background:**

Tissue factor (TF)-rich cancer microvesicles are correlated with thrombosis risk. Intrinsic coagulation factors are also associated with the risk of thrombosis in cancer patients. This study explored the roles of pancreatic cancer-derived microvesicles and intrinsic factors in thrombogenesis.

**Methods:**

Human pancreatic cancer cell lines rich in TF (AsPC-1-TF^high^, MIAPaCa-2-TF^high^) or poor in TF [AsPC-1-TF^KO^(knockout) and MIAPaCa-2-TF^low^] were generated for microvesicle preparation and injected into coagulation-defective mice. Inferior vena cava (IVC) clots and lung thrombosis were evaluated. Immunodeficient hemophilia A (NSG-HA) mice were orthotopically injected with the cells mentioned above, and the tumor and IVC clot weights were analyzed.

**Results:**

With the injection of TF^high^ microvesicles, IVC clots were rarely found in hemophilic mice. The TF^low^ and TF^KO^ microvesicles resulted in few IVC clots in any mouse. Lung thrombosis was substantially reduced in the hemophilic mice infused with any microvesicle type. In orthotopic tumor models, TF^high^ cells grew faster than did TF^low^ cells. TF^high^ tumor-bearing NSG-WT mice had the most enormous IVC clots, whereas NSG-HA mice had no IVC clots.

**Conclusion:**

Pancreatic cancer thrombosis induced by TF-expressing microvesicles strongly depended on FVIII and FIX, while VWF played a minor role. Moreover, TF, but not FVIII, was significantly related to tumor growth.

**Supplementary Information:**

The online version contains supplementary material available at 10.1186/s12959-025-00715-x.

## Background

Cancer-associated thrombosis (CAT), especially venous thromboembolism (VTE), is a major complication and the second leading cause of death among cancer-related deaths [1]. The incidence of CAT has been increasing over time, and this condition has become a substantial burden on cancer patients [2, 3]. Predicting factors for CAT [4–9] have been identified and include cancer site, stage, treatments, central intravenous catheters, body mass index, hemoglobin, white blood cell count, platelet count, and D-dimer.

Different cancers may have their unique mechanism inducing coagulation and subsequent VTE [10, 11]. Among all cancers, pancreatic cancer is recognized as the most thrombogenic [5]. In patients with pancreatic cancer, tissue factor (TF) on cancer cells [12], plasma [13], or circulating microvesicles (MVs) [13–15] is the major cause of thrombosis development. Mouse models have also shown that not only exogenous TF-expressing MVs [16] but also endogenous TF-expressing MVs released from pancreatic cancer cells growing in mice [17] can significantly promote thrombosis. Our recent report [9], which used a cohort of more than five thousand patients with lung cancer, pancreatic cancer, gastric cancer, and lymphoma, revealed that activated partial thromboplastin time (APTT) but not prothrombin time (PT) is significantly correlated with the risk of VTE. This finding indicates that the intrinsic pathway might also be involved in the CAT mechanism in lung cancer, pancreatic cancer, gastric cancer, and lymphoma.

In the modern model of coagulation processes, TF and factor VIII/IX are not entirely separated from each other, as in PT and APTT. The TF-FVIIa complex could activate factor X and factor IX. Tissue factor pathway inhibitor (TFPI) blocks the action of the TF-FVIIa complex, while the FVIIIa-FIXa complex is formed and activates factor X more efficiently.

An earlier study [18] showed that TF and factors VII, IX, VIII, and XII can be stained in pancreatic cancer cells and that local coagulation activation may regulate the growth of cancer cells. In addition, several studies have explored intrinsic pathway involvement in pancreatic cancer. One study [19] showed that mice orthotopically administered the TF-expressing pancreatic cancer cell line Bx-PC3 have similarly high thrombin-antithrombin levels, regardless of whether they were treated with antisense oligonucleotides against *F11* mRNA. However, TF-negative pancreatic cancer cells were not tested in this model. Another report [20] revealed that pulmonary embolism induced by MVs derived from pancreatic cancer cells is factor XII dependent in a mouse model. However, several clinical trials have demonstrated promising antithrombotic effects with drugs targeting Factor XI [21–23]. In contrast, factor XII antagonists have not yet been evaluated as antithrombotic therapies [24, 25].

Nevertheless, two additional factors, factors VIII (FVIII) and IX (FIX), are involved in APTT but not in PT; these factors also play important roles in hemostasis and thrombosis, but they have yet to be thoroughly studied in CAT. In addition, FVIII binds to von Willebrand factor (VWF). High VWF was also found to be associated with thrombosis risk [26]. Some patients with von Willebrand disease (VWD) also have low FVIII [27], while FVIII and VWF often increase simultaneously. Therefore, it isn’t easy to differentiate the contribution of the prothrombotic effect between FVIII and VWF in clinical studies. Our VWD mouse model [28] with *VWF* R1326H mutant knock-in, which disables the binding between VWF and platelets without reducing FVIII, is suitable for this study.

This study aimed to reveal whether the factors mentioned above, including FVIII, FIX, and VWF, affect pancreatic cancer-related thrombosis, specifically that induced by pancreatic cell-derived MVs, with or without TF, in mouse models. Additionally, we aimed to study whether FVIII and TF affect the growth of pancreatic cancer. Immune-deficient mice [29–31] are a good model for evaluating the growth of human pancreatic cells without rejection from the mouse immune system.

Methods and materials.

### Pancreatic cancer cell lines

See the “Supplemental methods” section for details. Two mycoplasma-free human pancreatic cell lines were used in this study: AsPC-1 (Bioresource Collection and Research Center, Taiwan, BCRC no. 60494), which has high TF expression; AsPC-1 (TF^high^); MIA PaCa-2 (Bioresource Collection and Research Center, Taiwan, BCRC no. 60139), which has almost undetectable TF expression; and MIAPaCa-2 (TF^low^). CRISPR/Cas9 technology was applied to generate the AsPC-1 TF knockout cell line AsPC-1 (TF^KO^). TF-overexpressing MIA PaCa-2 cells were generated via the transfection of the parental MIA PaCa-2 cell line with a TF expression plasmid (see supplemental method), and the resulting cell line was defined as MIA PaCa-2 (TF^high^). The parental AsPC-1 (AsPC-1-TF^high^) and CRISPR-derived AsPC-1-TF^KO^ cells were cultured in Roswell Park Memorial Institute (RPMI) 1640 medium. The parental MIA PaCa-2 (TF^low^) and TF-overexpressing MIAPaCa-2-TF^high^ cells were maintained in Dulbecco’s modified Eagle’s medium (DMEM), and both culture media were supplemented with 10% fetal bovine serum and 1% streptomycin/penicillin.

### Isolation of MVs and nanoparticle tracking analysis (NTA)

To collect MVs from cells [32], 1 × 10^8^ cells/roller bottle were seeded and cultured for 5 days. The medium was then replaced with a serum-free medium, and the cell suspension was collected after 24 h; this process was repeated for five consecutive days. After 5 days of collection, we performed the first centrifugation at 2612 × *g* for 5 min at 4 °C (JA-14 rotor, Optima L-100 K, Beckman Coulter, Inc.) to remove debris and the second centrifugation at 20,000 × g for 20 min at 4 °C (JA25.5 rotor, Avanti J-26XP, Beckman Coulter, Inc.). Then, 1% HBSA (137 nM NaCl, 5.38 mM KCl, 5.55 mM glucose, 10 mM HEPES, and 0.1% BSA, pH = 7.5) was used to collect the MV pellets, and a bicinchoninic acid protein assay (BCA) was used to determine the protein concentration. The MV concentration and size distribution were measured via a NanoSight LM10-HS (Malvern Instruments), and NTA was conducted at the Center for Micro/Nano Science and Technology of National Cheng Kung University. Briefly, the scattered light from liquid suspension nanoparticles under Brownian motion is directly captured through a microscope lens to obtain high-resolution particle size distribution and nanoparticle concentration information.

### Transmission electron microscopy (TEM)

For MV images, the MV suspension (in 1% HBSA) was centrifuged at 20,000 × g for 20 min at 4 °C via a refrigerated microcentrifuge model 3520 (Kubota Co., Ltd., Japan). Then, a 1.5% agarose gel was used to immobilize the MV pellet, which was stored in fixation buffer. Slice manufacturing and TEM image processing (JEM-1400, JEOL, Ltd., Japan) were conducted by the Department of Pathology of National Taiwan University Hospital.

### TF activity assay of MVs

We used Dade Innovin (Siemens, Marburg, Germany), which contains 331 ng/ml [33] recombinant human TF protein, as a standard, and phospholipid and Ca^2+^ were used to make serial dilutions of Dade Innovin. A Sysmex CA50 Coagulation Analyzer (CA-50) was subsequently used to test the PT of the standard and its serial dilutions to obtain a standard curve. The MV samples were also diluted with phospholipid and Ca^2+^, and by testing the PT of the MV samples, we obtained the corresponding MV-TF concentration.

### Western blot of MVs

Immunoblotting was performed as described previously [34]. An appropriate amount of cell lysates and MV preparation (20 µg of total protein) was resolved in cracking buffer dye (150 mM Tris-HCl pH = 6.8, 6% SDS, 0.01% bromophenol blue, 30% glycerol, and 10% β-mercaptoethanol), boiled for 10 min, subjected to sodium dodecyl sulfate‒polyacrylamide gel electrophoresis (SDS‒PAGE), transferred to a PVDF membrane and detected by an anti-human TF antibody (Abcam #ab211016). We used human CD81 as a marker for microvesicles [35]. Anti-human CD81 antibody (Abcam #ab59477) detection should be conducted under nonreducing conditions. β-Mercaptoethanol was not added to the cracking buffer dye, and the samples were not boiled when we performed anti-human CD81 antibody detection, while the other procedures were the same as those described above. The PVDF membrane was blocked with 5% nonfat milk in TBST (0.5% Tween 20, 150 mM NaCl, 20 mM Tris-HCl, pH = 7.5). The secondary antibodies used were HRP-conjugated anti-rabbit IgG (Abcam, 1:2000) and anti-mouse IgG (Abcam, 1:2000). Protein detection was performed via a chemiluminescent system (Millipore, MA, USA) and an ImageQuant LAS4000 equipped with Multigauge software (Fujifilm, Tokyo, Japan). α-Tubulin and β-actin were used as internal controls.

### Flow cytometric analysis of MVs

The MV suspension samples were centrifuged at 20,000 × g for 20 min at 4 °C via a refrigerated microcentrifuge model 3520 (Kubota Co., Ltd., Japan), after which the supernatant was removed to collect the MV pellets. The pellets were resuspended in 0.1 μm filter-treated PBS, and APC-conjugated anti-human TF antibody (BioLegend, Inc., USA #365205) was added for a 1-h incubation. Megamix-Plus SSC Counting Beads (Biocytex) were used to establish the proper size range (160–500 nm) of MVs, measured by an Attune™ NxT flow cytometer.

### Mouse models

The Institutional Animal Care and Use Committee (IACUC) reviewed and approved all procedures at the National Taiwan University College of Medicine Laboratory Animal Center (NTUCMLAC). *F8* knockout [36] mice (hemophilia A, HA) were originally supplied by Dr. H. H. Kazazian Jr., and *F9* knockout [37] mice (hemophilia B, HB) were kindly provided by Dr. Darrel Stafford from the University of North Carolina at Chapel Hill. The *VWF* R1326H mutant knock-in [28] (VWD) model was generated on a C57BL/6 mouse background as described previously. Each strain was bred in-house and backcrossed for 10 generations with C57BL/6 mice. *VWF* R1326H disables murine VWF binding to GPIb/IX on platelets, which creates conditions similar to those of VWD type 2 M. Immunodeficient (NOD. Cg-*Prkdc*^*scid*^*Il2rg*^*tm1Wjl*^/SzJ, NSG) mice [30, 31] were used to evaluate tumor growth. Immunodeficient hemophilia A mice (NOD. Cg-Prkdc^scid^ Il2rγ^tm1Wjl^/SzJ; F8^em1^/swl, NSG-F8^em1^/swl) were generated as previously described [29, 38]. Wild-type littermate mice were used as controls.

### MV-induced thrombosis mouse model

We applied IVC stenosis to induce thrombosis in the IVC [16]. First, 8- to 28-week-old male mice (weighing 25–35 g) were anesthetized via intraperitoneal injection of 2.5% avertin, and the mice were kept supine. After laparotomy, all side branches were tied completely, and then, the IVC was ligated with a 26G needle as a spacer to create partial flow restriction. The spacer was removed after IVC ligation. After IVC stenosis was achieved, 40 µg of MV was injected intravenously into the mice. The mice were sacrificed 3 h later, and the clot weight within the IVC and thrombi within the vessels of the lungs were measured. Specifically, lung tissue sections stained with hematoxylin and eosin (H&E) were examined under a 400X Olympus CH20 microscope (Olympus Corp., Japan). The number of thrombi within vessels from twenty random observation fields was calculated and is summarized as the representative outcome of lung thrombosis.

### MV staining of clots and lung tissue

Clots and lung tissue were collected from the mice, fixed with 10% formaldehyde, embedded in paraffin wax, and then cut into 5-µm thick sections. The sections were incubated in boiled citrate buffer for 10 min to retrieve the antigen, followed by blocking with 10% normal goat serum for 2 h. The MVs were stained with rabbit anti-human CD81 antibody (iReal Biotechnology) followed by Alexa Fluor^®^ 488 conjugated goat anti-rabbit antibody, and the nuclei were stained with DAPI (Dako). Images of the sections were captured via an inverted microscope (TS100-F, Nikon, Tokyo, Japan).

### Tumor-bearing mouse model

AsPC-1 (TF^high^), AsPC-1 (TF^KO^), MIA PaCa-2 (TF^low^), and MIA PaCa-2 (TF^high^) cells were isolated for orthotopic pancreatic injection (1 × 10^6^ cells in 50 µL of PBS) in 10- to 28-week-old male NSG mice and NSG-HA mice. Five weeks after the cancer cell injection, we applied IVC stasis for 1 h to stimulate thrombosis, then euthanized the mice to assess clot weight within the IVC and tumor growth.

### Statistical analysis

All the statistical analyses were performed with GraphPad Prism 6.0 (GraphPad Software, CA, USA). An unpaired nonparametric Mann- Whitney U test was used for clot weight, lung occlusions, and tumor weight analyses, and a P value < 0.05 was considered to indicate statistical significance.

## Results

### Cell lines and MV characteristics

This study utilized four human pancreatic cell lines characterized by high or low TF content. Specifically, the AsPC-1-TF^high^ and MIAPaCa-2-TF^high^ cell lines are rich in TF, whereas the AsPC-1-TF^KO^ and MIAPaCa-2-TF^low^ cell lines are poor in TF. These cell lines were analyzed for TF expression and evaluations of cell proliferation and migration (Supplementary Figures S1-S4). The findings indicated that the differences in cell proliferation and migration are not statistically significant when comparing the AsPC-1 parent cell line to its derivatives (AsPC-1-TF^high^ versus AsPC-1-TF^KO^) or the MIAPaCa-2 parent cell line to its derivatives (MIAPaCa-2-TF^low^ versus MIAPaCa-2-TF^high^).

**Fig. 1:**
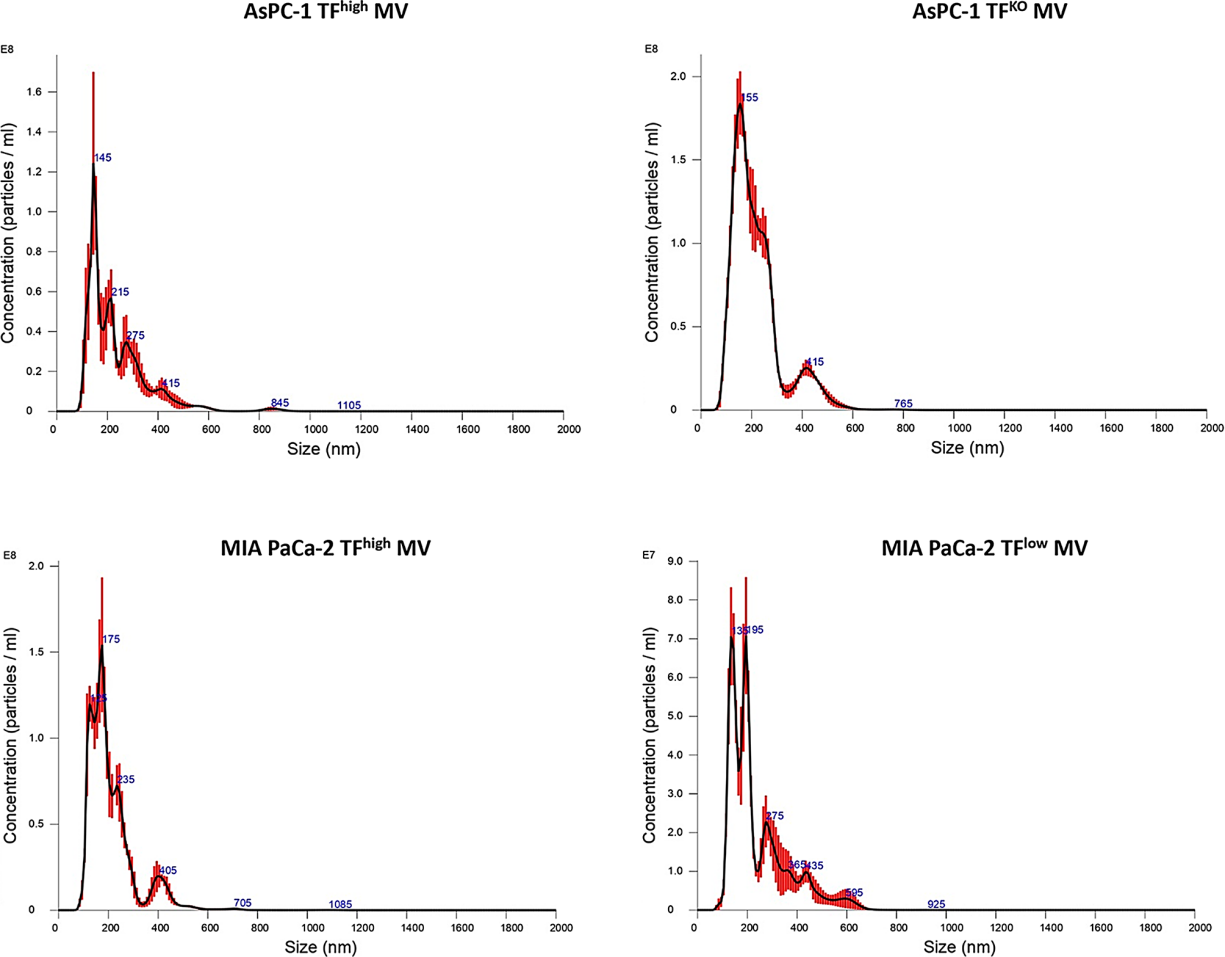
Size distribution of MVs derived from AsPC-1, MIA PaCa-2 cells was conducted using nanoparticle tracking analysis. Representative histograms are shown

**Fig. 2:**
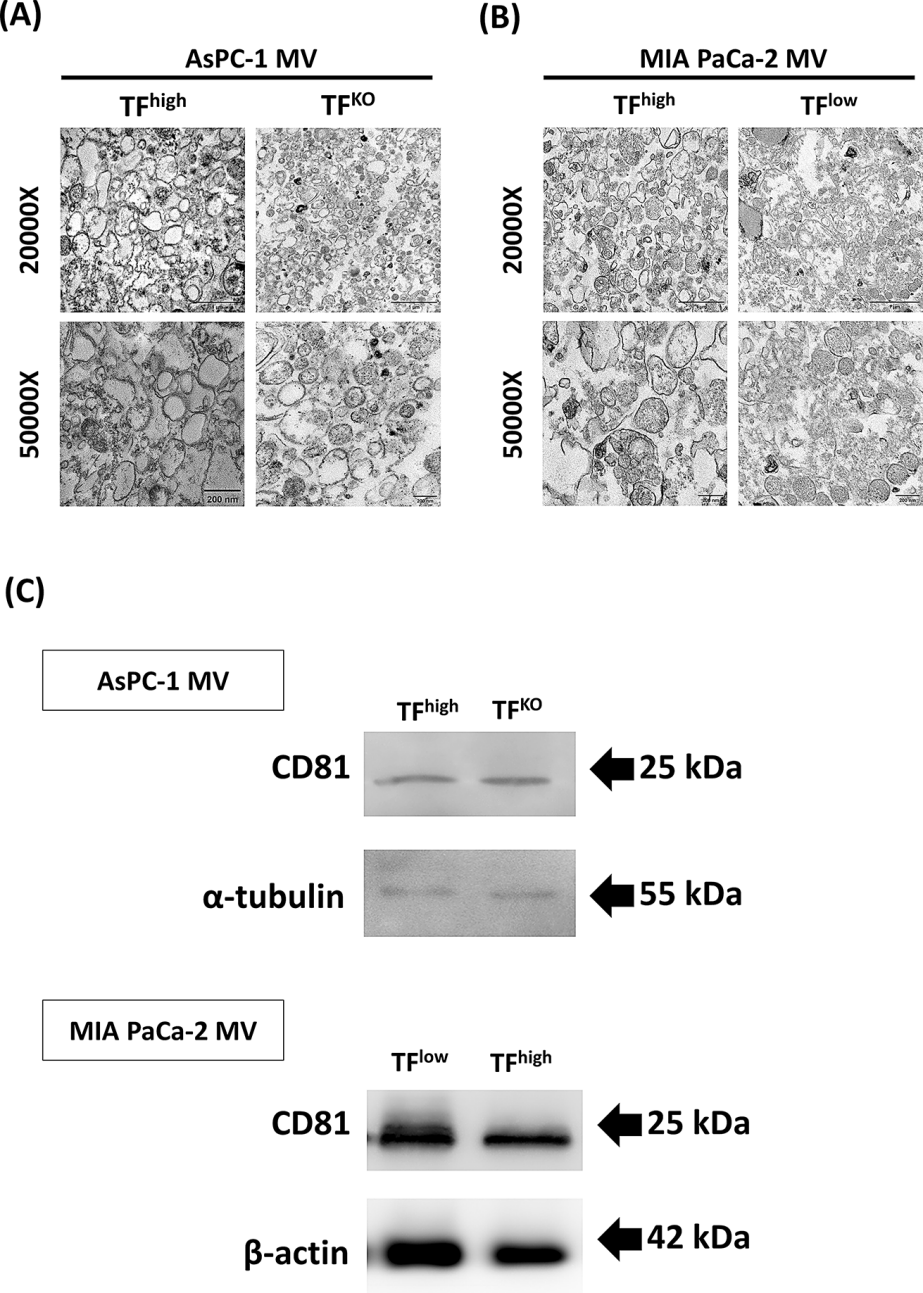
(**A**, **B**) Structure of MVs derived from (A) AsPC-1 (TF^high^) and AsPC-1 (TF^KO^) and (**B**) MIA PaCa-2 (TF^high^) and MIA PaCa-2 (TF^low^), imaged by transmission electron microscopy at 20,000x and 50,000x magnification. Scale bars: 1 μm and 200 nm, respectively. (**C**) Western blot analysis of CD81 expression in MVs derived from AsPC-1 and MIA PaCa-2 cells (20 µg/well). α-Tubulin and β-actin were used as internal controls

Different MVs were prepared from four human pancreatic cancer cell lines, AsPC-1 (TF^high^), AsPC-1 (TF^KO^), MIA PaCa-2 (TF^high^), and MIA PaCa-2 (TF^low^), and the sizes of the MVs generated from these cell lines were 65–1265 nm (median of 233.9), 45–1005 nm (median of 218.4), 65–1255 nm (median of 166.5), and 65–1125 nm (median of 173.2), respectively. Among all the MV preparations, 93–99% were within the proposed size range (100–1000 nm), as shown in Fig. 1. All types of MVs presented intact phospholipid membranes, as shown by TEM (Fig. 2A and B). The expression of the MV surface marker CD81 on the isolated MVs was also confirmed by western blotting (Fig. 2C).

### TF measurement of MVs

**Fig. 3:**
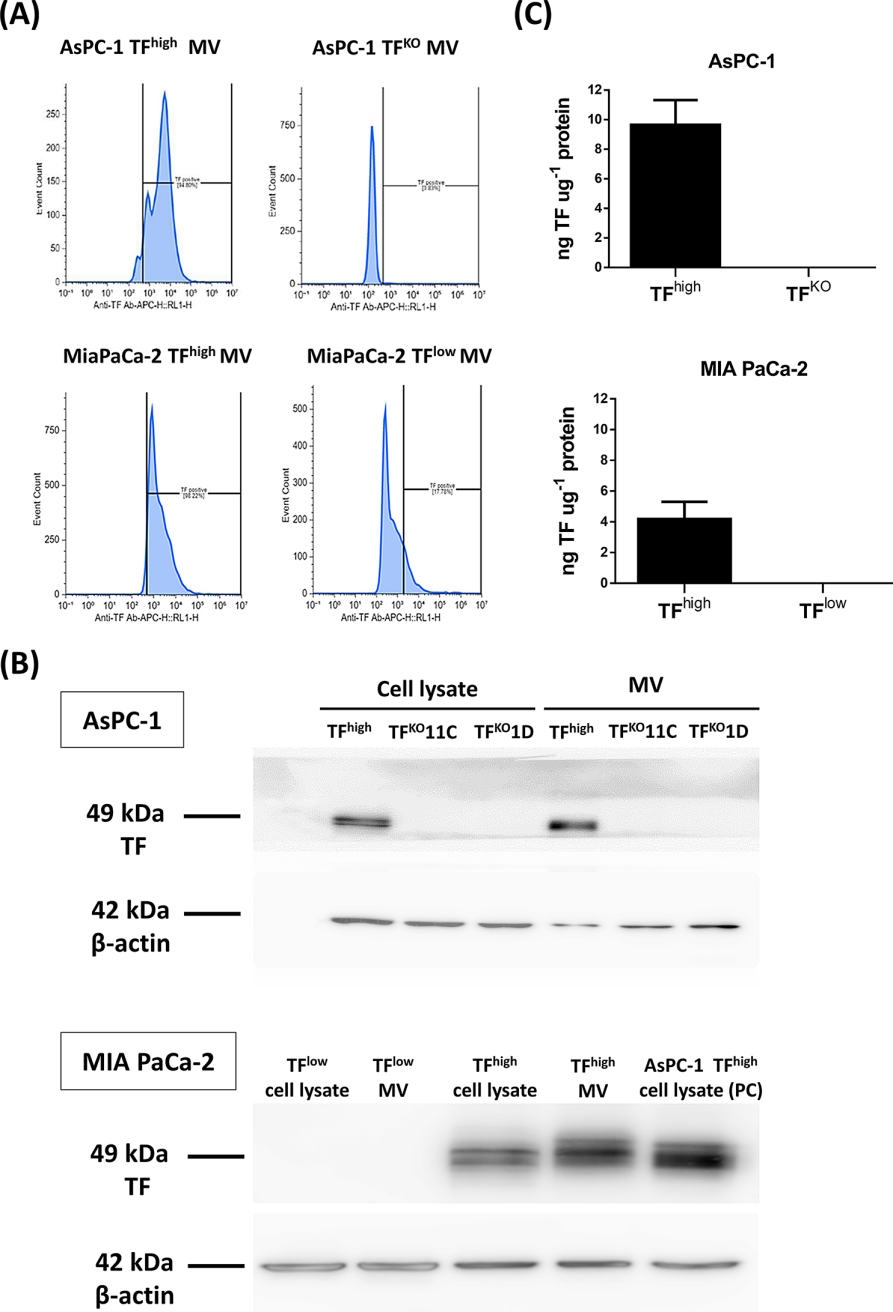
(**A**) Expression of TF on MVs was determined using an APC-conjugated anti-human TF antibody by flow cytometry, with gating based on an unstained sample (data not shown). The horizontal axis represents TF-positive cells, and the vertical axis represents cell count. (**B**) Western blot analysis of TF expression in AsPC-1 and MIA PaCa-2 cells and MVs (20 µg/well). AsPC-1 TF^high^ cell lysate (PC) was used as positive control of TF expression and β-actin was used as internal control. (**C**) TF activity was measured using a 1-stage clotting assay

The TF expression and activity of the cell line-derived MVs were examined, and the results were consistent with the expected TF genotypes. According to flow cytometry, TF was detected in 94.8% of AsPC-1 (TF^high^)-generated MVs and 98.22% of MIA PaCa-2 (TF^high^)-generated MVs, whereas TF was detected in only 0.83% of AsPC-1 (TF^KO^)-generated MVs and 17.8% of MIA PaCa-2 (TF^low^)-generated MVs (Fig. 3A). Using western blotting, we confirmed that both AsPC-1 (TF^high^)- and MIA PaCa-2 (TF^high^)-derived MVs were positive for TF, whereas MVs derived from both AsPC-1 (TF^KO^) and MIA PaCa-2 (TF^low^) cells had no detectable TF (Fig. 3B). TF is a glycosylated protein; therefore, multiple bands are observed on Western blotting. With a protein concentration of 0.4 µg/µL of MVs derived from AsPC-1 (TF^high^) and MIA PaCa-2 (TF^high^) cells, the TF functional assay results revealed 9.66 ng/mL and 4.18 ng/mL relative TF activity, respectively. The other two MVs had no detectable TF activity (Fig. 3C).

### TF-negative MVs and hemophilic mice show independent reductions of thrombosis

**Fig. 4:**
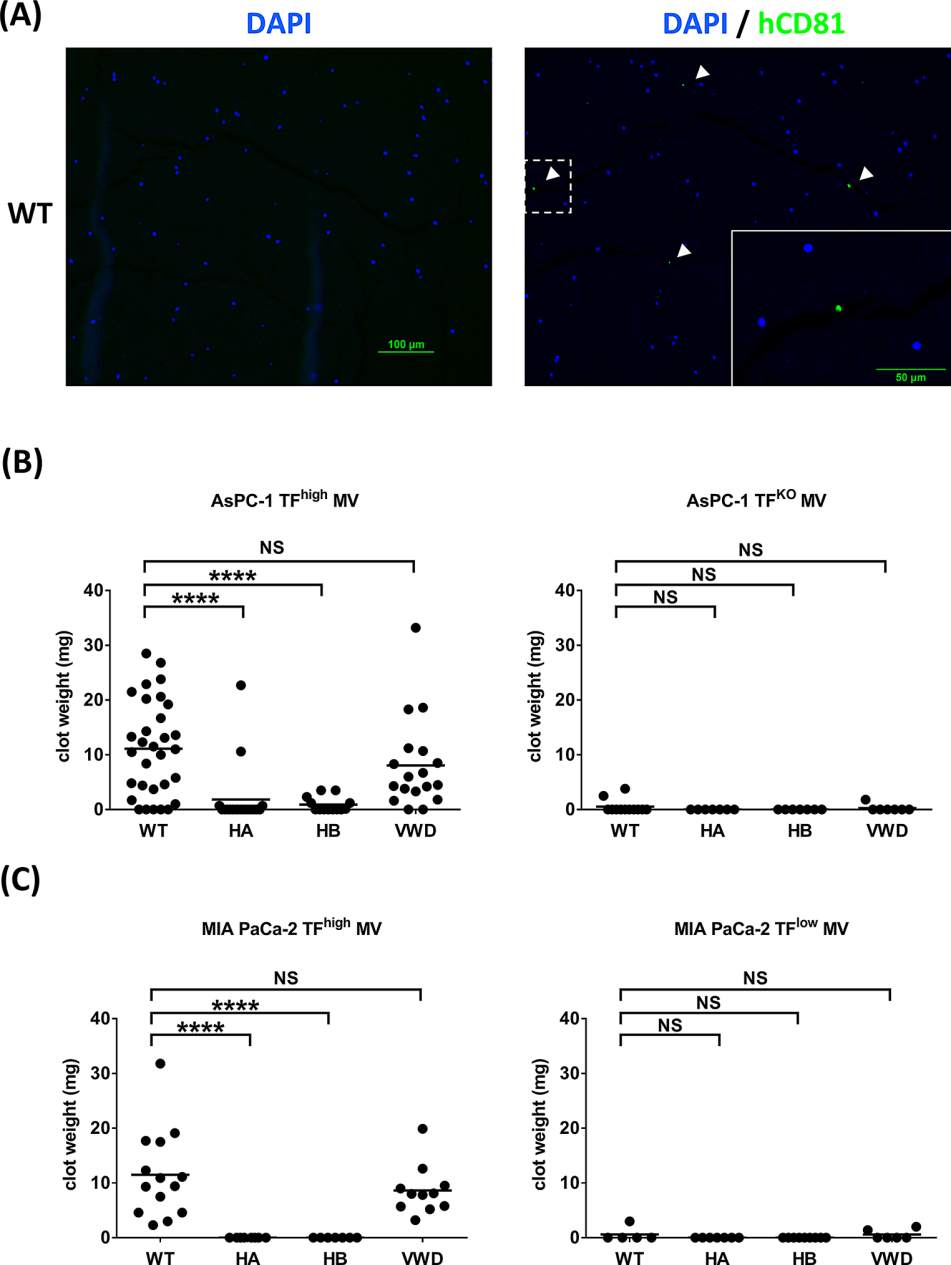
(**A**) Clot in the IVC model of WT mice injected with MIA PaCa-2 TF^high^ MVs, in which the MVs were stained with rabbit anti-human CD81 antibody followed by Alexa Fluor^®^ 488 conjugated goat anti-rabbit antibody and analyzed by immunofluorescence. Nuclei were stained with DAPI. Scale bar, 100 μm. Insert, magnification of the dashed box. Scale bar, 50 μm. The left panel shows staining with DAPI alone as a control, while the right panel displays staining with Anti- human CD81 antibody. (**B**) After IVC stenosis was achieved, 40 µg of MV from AsPC-1 (TF^high^/TF^KO^) cells were injected intravenously into the WT (*n* = 31/12), HA (*n* = 19/7), HB (*n* = 13/7) and vWD mice (*n* = 18/7); (**C**) After IVC stenosis was achieved, 40 µg of MV from MIA PaCa-2 (TF^high^/ TF^low^) cells were injected intravenously into the WT (*n* = 14/5), HA (*n* = 8/7), HB (*n* = 7/9) and vWD mice (*n* = 11/6). Each dot represents one mouse, with a line indicating the mean. * *p* ≤ 0.05; ** *p* ≤ 0.01; *** *p* ≤ 0.001; **** *p* ≤ 0.0001; NS, non-significant

After the injection of MVs into mice followed by IVC stenosis, fluorescent staining shows a very small amount of CD81 expression, suggesting the potential presence of MVs within the IVC clot. (Fig. 4A) Analysis of the IVC clot weights revealed that with the injection of MVs from AsPC-1 (TF^high^) and MIA PaCa-2 (TF^high^) cells, WT and VWD mice formed similar amounts of clots, whereas HA and HB mice had almost no IVC clots (Fig. 4B and C). With the injection of MVs derived from AsPC-1 (TF^KO^) and MIA PaCa-2 (TF^low^) cells, all the mice, including the WT mice, had almost no IVC clots.

**Fig. 5:**
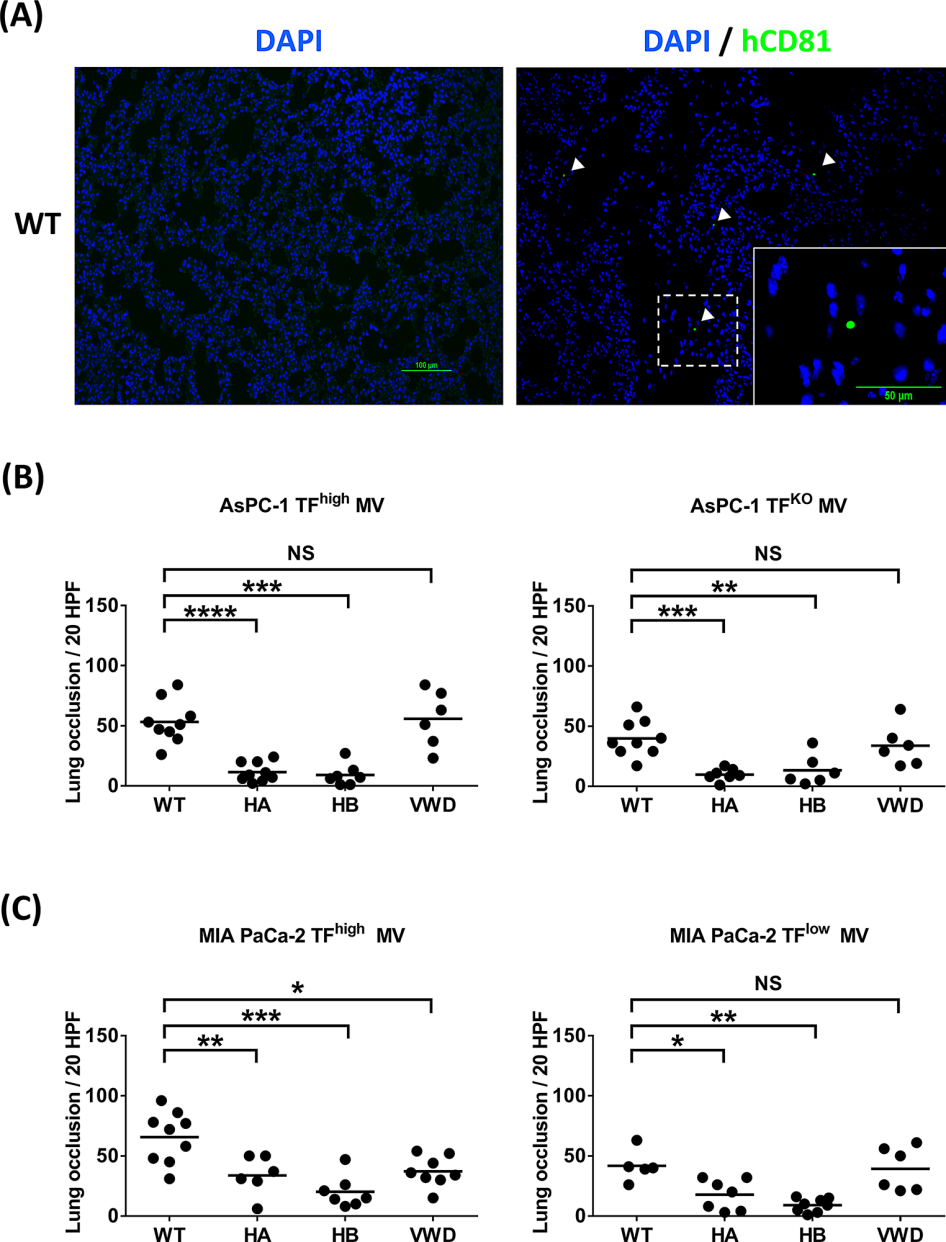
(**A**) Lung tissue sections from the IVC model of WT mice injected with MIA PaCa-2 TF^high^ MVs, in which the MVs were stained with rabbit anti-human CD81 antibody followed by Alexa Fluor^®^ 488 conjugated goat anti-rabbit antibody and analyzed by immunofluorescence. Nuclei were stained with DAPI. The left panel shows staining with DAPI alone as a control, while the right panel displays staining with Anti- human CD81 antibody. Scale bar, 100 μm. Insert, magnification of the dashed box. Scale bar, 50 μm. (**B**, **C**) Lung tissue sections stained with H&E were examined at 400x magnification. The number of thrombi within vessels was counted from twenty randomly selected observation fields, and the results are summarized as the representative measure of lung thrombosis. Each dot represents one mouse, with *n* = 5–9 per group. Lines in dot plots represent the mean. * *p* ≤ 0.05; ** *p* ≤ 0.01; *** *p* ≤ 0.001; **** *p* ≤ 0.0001; NS, not significant

To investigate whether TF-positive and TF-negative MVs form thrombi in tissues and organs independent of FVIII or FIX, we examined mouse lungs, and thrombi within lung vessels after the injection of four different MVs into mice with IVC stenosis were identified (Supplemental Figure S6 and Supplemental Table S1). Figure 5A shows a very small amount of CD81 expression, suggesting the potential presence of MVs within the lung thrombi. After the injection of MVs derived from AsPC-1 cells (TF^high^), the WT and VWD mice presented similar numbers of thrombi, whereas the HA and HB mice presented significantly fewer but still some lung thrombi. However, after injection of MVs from MIA PaCa-2 (TF^high^) cells, WT mice presented the greatest number of lung thrombi, VWD mice presented a slightly lower number, and HA and HB mice presented the lowest number. With the injection of both TF-negative MVs derived from AsPC-1 (TF^KO^) and MIA PaCa-2 (TF^low^) cells, WT and VWD mice presented equally high numbers of lung thrombi, whereas HA and HB mice presented significantly lower numbers. There was no significant difference in the number of lung thrombi between HA and HB mice (Fig. 5B and C).

### TF supports tumor growth and IVC clots, but FVIII supports only IVC clots

**Fig. 6:**
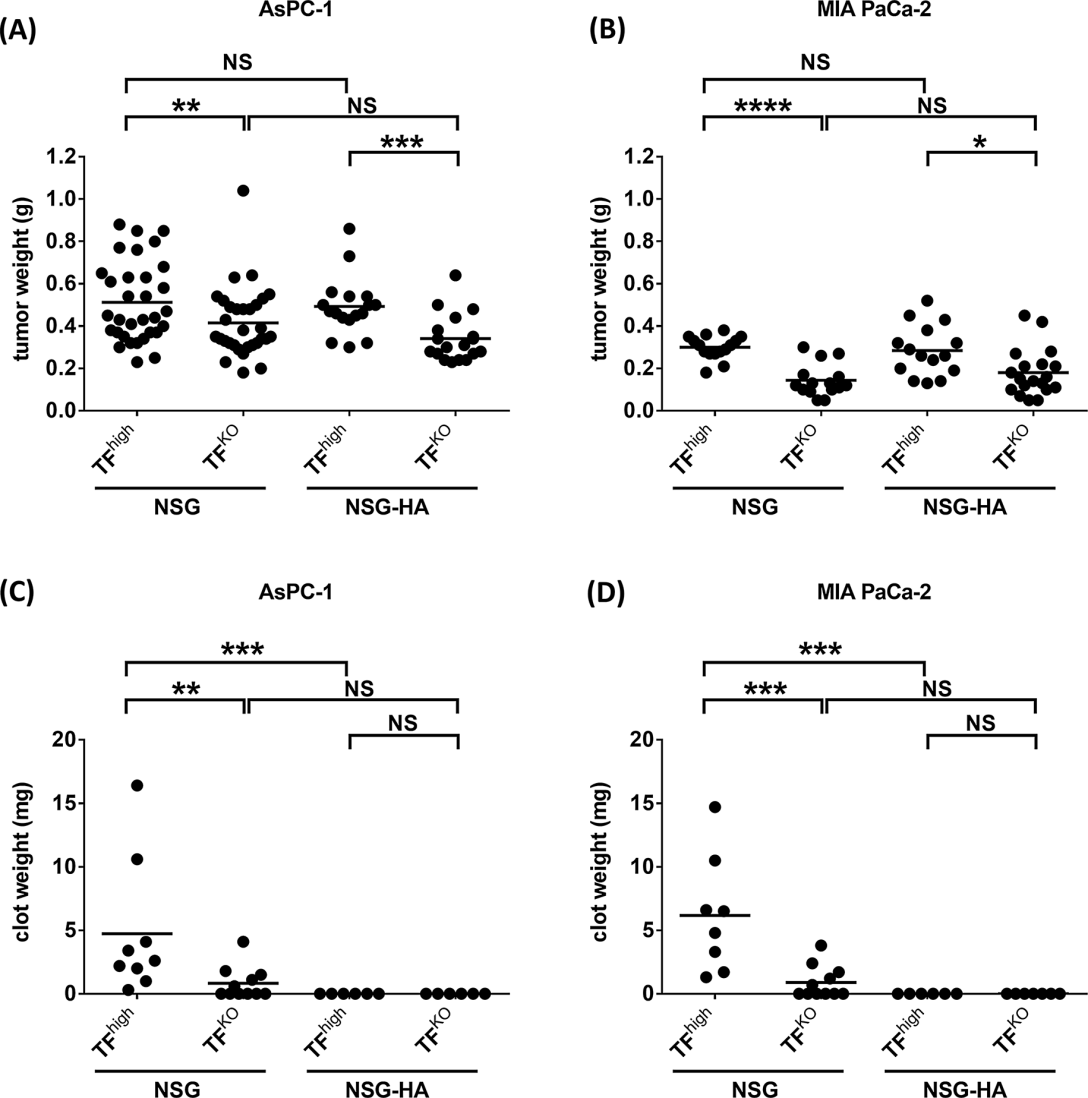
TF supports tumor growth and IVC clots. 1 × 10^6^ AsPC-1 (TF^high^/TF^KO^) and MIA PaCa-2 (TF^high^/TF^low^) cells were orthotopically injected into the pancreas of NSG and NSG-HA mice. After 5 weeks, IVC flow was completely blocked for 1 h, and the mice were sacrificed to measure (**A**, **B**) tumor weight and (**C**, **D**) clot weight. * Each dot represents one mouse, with *n* = 6–32 per group. Lines in dot plots represent the mean. *p* ≤ 0.05; ** *p* ≤ 0.01; *** *p* ≤ 0.001; **** *p* ≤ 0.0001; NS, non-significant

Four different pancreatic cancer cell lines were orthotopically injected into NSG and NSG-HA mice for 5 weeks, and representative images of the tumors are shown in Fig. 6A and B. We found that AsPC-1 (TF^high^) cells grew significantly faster than its TF-negative control, AsPC-1 (TF^KO^) cells (Fig. 6C), whereas MIA PaCa-2 (TF^low^) cells grew significantly slower than its TF-overexpressing counterpart, MIA PaCa-2 (TF^high^) cells (Fig. 6D). However, the growth of tumors derived from the same cell line did not differ between NSG and NSG-HA mice.

Clot weight within the IVC was measured after IVC stasis was induced in tumor-bearing NSG and NSG-HA mice, as shown in Fig. 6E and F. With respect to TF-positive cancer cells, AsPC-1 (TF^high^) or MIA PaCa-2 (TF^high^) NSG mice had significantly more IVC clots than NSG-HA mice did. Only a few TF-negative cancer cell-bearing NSG mice had IVC clots, which were significantly fewer than those of TF-positive cancer cell-bearing NSG mice. Almost none of the NSG-HA mice exhibited IVC clot formation, regardless of the cancer cell type. These results are consistent with those of MV-evoked IVC stenosis models in which both TF and FVIII are critical for the formation of IVC clots.

## Discussion

In this study, we used two different pancreatic cancer cell lines, AsPC-1, which was originally characterized by TF expression, and MIA PaCa-2, which was characterized by low TF expression. With these cell lines, we generated AsPC-1 (TF^KO^) and MIA PaCa-2 (TF^high^) cells as counterpart controls to explore pancreatic cancer cell-derived MVs and cancer cell-induced thrombosis, as well as tumor growth, in different mouse models. Our results revealed that TF, FVIII, and FIX, but not VWF, correlated with cancer cell-derived MV-induced thrombosis. Both TF and FVIII correlated with thrombosis induced by orthotopically injected cancer cells, but most importantly, TF is the only factor that increases tumor growth.

TF is known to play important roles in pancreatic cancer cell-induced thrombosis. Unsurprisingly, TF-positive MVs derived from AsPC-1 (TF^high^) or MIA PaCa-2 (TF^high^) cells induced more IVC and more lung thrombi than did TF-negative MVs derived from AsPC-1 (TF^KO^) or MIA PaCa-2 (TF^low^) cells in WT mice. Neither FVIII (HA mice) nor FIX (HB mice) resulted in IVC clot formation but reduced lung thrombi after various MV injections. In addition, mice that were infused with TF-negative MVs exhibited nearly no IVC clots. We believe that a lack of FVIII, FIX or TF could significantly reduce the thrombosis load; hence, most of the thrombi were carried by the bloodstream to the lung vessels, and few remained within the IVC. Summarizing the results of IVC clot and lung vessel occlusion, we found that the combination of FVIII/FIX deficiency and TF-negative MV injection clearly induced the lowest total thrombosis load. In contrast, VWD model mice presented minimal differences in terms of clot formation compared with WT mice. These findings demonstrate that FVIII and FIX are involved in MV-induced thrombosis independent of TF, whereas VWF has a limited role in the thrombotic process. These results are consistent with the findings in our cohort [9] and previous studies on TF in pancreatic cancer [12, 15].

In the tumor-bearing NSG and NSG-HA mouse experiments, TF^high^ pancreatic cancer cells grew faster than TF^KO^ or TF^low^ cells did in both the NSG and NSG-HA mice, whereas the growth of the same cancer cells within the NSG and NSG-HA mice did not differ. These findings indicate that TF, but not FVIII, is correlated with the tumor growth of cancer cells.

Clinical observations have shown that cancer patients with circulating TF-positive MVs may have a greater risk of thrombosis and poorer survival [14, 15]. Cancer patients with VTE also have poorer survival [3, 9, 39], and VTE-related death is not the only cause of this difference. Thrombotic events could also serve as an independent parameter for increased cancer-related mortality. This observation may be explained by TF expression being increased by cancer-driving mutations, such as mutations in TP53 or KRAS, in colorectal cancer cells [40] and non-small cell lung cancer cells [41]. However, Wojtukiewicz’s group reported that TF as well as factors VII, IX, VIII, and XII were present on resected pancreatic tumor cells and it was suggested that fibrin deposited around tumor cells might regulate tumor growth [18]. Clotting-dependent pathways involve the binding of FVIIa to the extracellular domain of TF, leading to the activation of the extrinsic coagulation cascade [42, 43]. Additionally, Ruf’s group reported that the TF-VIIa protease complex, which is independent of coagulation activation, can stimulate tumor and developmental angiogenesis through signaling via protease-activated receptor-2 (PAR-2) [44, 45]. These results may support our finding that only TF but not FVIII is related to tumor growth. In our study, we changed only the TF status without manipulations of known oncogenes, and we showed that cancer cells with TF expression grew faster than those without TF. These findings indicate that TF may increase pancreatic cancer growth and the process could be independent to its coagulation role. Taken together, we conclude that while FVIII is significantly involved in cancer associated thrombosis but not in the growth of cancer, TF affects both.

From the clinical point of view, although many observations revealed poorer survival of cancer patients with VTE than those without VTE [3, 9, 39], anticoagulants, which can successfully treat [46, 47] or prevent VTE [48–50], did not demonstrate any significant improvement of overall survival. These findings are consistent with our results that alleviation of hypercoagulability, either by the application of anticoagulants or FVIII deficiency, has no effect on reducing the growth of cancer itself. Further studies may explore whether the inhibition of TF or the TF-FVIIa complex could yield any anticancer effects while reducing thrombosis formation.

In conclusion, we used two different pancreatic cancer cell lines and two generated cell lines with opposite TF statuses as comparable controls in this study. We found that TF, FVIII, and FIX are related to cancer cell-derived MV-induced thrombosis, whereas VWF plays a very limited role in this process. Similarly, both TF and FVIII are related to thrombosis induced by orthotopically injected cancer cells. However, TF is the only factor that increases tumor growth.

## Electronic supplementary material

Below is the link to the electronic supplementary material.

Supplementary Material 1

## Data Availability

No datasets were generated or analysed during the current study.
