# Supplementary material for: Tissue factor, factor VIII and IX in microvesicle-induced thrombosis and tumor growth of pancreatic cancer
Source: Thromb J. 2025 Apr 11;23:32. doi: 10.1186/s12959-025-00715-x (PMC11987238; doi:10.1186/s12959-025-00715-x)
Supplement: Supplementary file 1 — Supplementary Material 1 [file 12959_2025_715_MOESM1_ESM.docx]

**Supplementary materials**

**Supplementary methods**

AsPC-1(TF^KO^)

To generate a TF-null AsPC-1 subline, the CRISPR/Cas9 system was applied with the help of the Biomedical Resource Core at the First Core Labs of National Taiwan University College of Medicine. Two single-guide RNAs (sgRNAs) were designed to target exon1 and exon 6 of TF, respectively, to delete a 11 kb region and result in loss of TF expressing. Four AsPC-1-TF^KO^ clones were confirmed by PCR the genomic DNA for the TF gene followed by sequencing of the PCR products with the Sanger method. Loss of TF expression was confirmed by Q-RT-PCR for absence of TF RNA and by western blot for absence of TF protein.

MIA PaCa-2 (TF^high^)

The TF cDNA was obtained by reversed transcription (RT)-PCR using total RNA extracted from AsPc-1 cells, sequenced by the Sanger method, inserted into the pCDNA3.1 plasmid and under the control of the CMV promoter. The resultant TF expression plasmid was delivered into MIA PaCa-2 cells through liposome-based transfection (lipofectamine 3000) according to the manufacturer’s instruction. Twelve TF expressing stable clones were isolated by resistance to G418 antibiotics. All clones were analyzed for RNA expression by quantitative RT-PCR and for protein expression by western blotting using human TF antibody (Abcam).

MTT assay

Cells were seeded in 96-well plates, at a density of 1x10^4 cells/well and cultured overnight at 37°C in a 5% CO2 incubator. After incubation for 24 hr, each well was treated with 10 μl of 5 mg/ml MTT reagent according to manufacturer’s instructions (Roche cell proliferation kit I). Live cells oxidize the MTT reagent through the succinate dehydrogenase (SDH) in the mitochondria, leading to a redox reaction that produces purple Formazan crystals. After MTT treatment for 4 hr, each well was treated with DMSO to dissolve the purple crystals, and measured at OD 570nm for assess of cell viability and proliferation capability. Standard curve was prepared according to the manufacturer’s instructions (Roche cell proliferation kit I).

Wound healing assay

A method similar to scratch wound assay was used to analyze cell migration activity. Basically, cells were seeded in a 6-well plate inserted with cell culture inserts (ibidi IB-80206) at a density of 1x10^5 cells/70 μl per well into each insert, and cultured overnight at 37°C in a 5% CO2 incubator. After culture for 24 hr, cell confluency within the grid was confirmed under a microscope and cell culture insert was removed using a forceps. Cell-free spaces generated by the cell culture insert in each well was confirmed under the microscope. Migration of cells into the cell free-space was imaged manually every 4 hours using an optical microscope (Olympus). Three repetitions were performed for each time point. The migration distance was measured using the Image J software and used as a representative of the wound healing activity of cells.

**Supplementary figures**


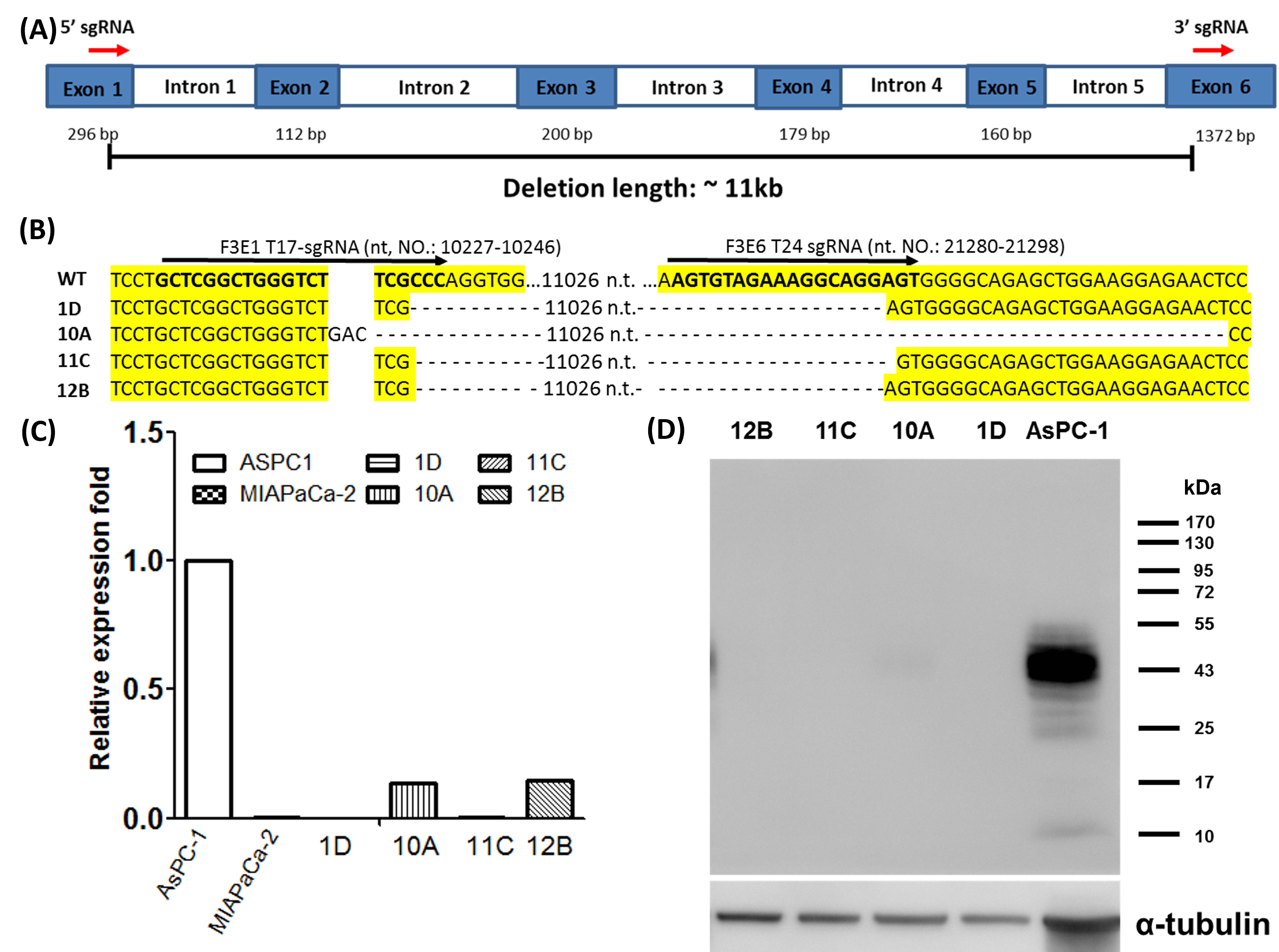


Figure S1: Generate AsPC-1 TF-knockout cell line (TF^KO^) (A) Two single-guide RNAs (sgRNAs) were designed to target exon1 and exon 6 of TF, respectively, to delete a 11 kb region and result in loss of TF expressing. (B) Four AsPC-1-TF^KO^ clones were confirmed by PCR the genomic DNA for the TF gene followed by sequencing of the PCR products with the Sanger method. (C, D) Loss of TF expression was confirmed by Q-RT-PCR for absence of TF RNA(C) and by western blot for absence of TF protein(D).


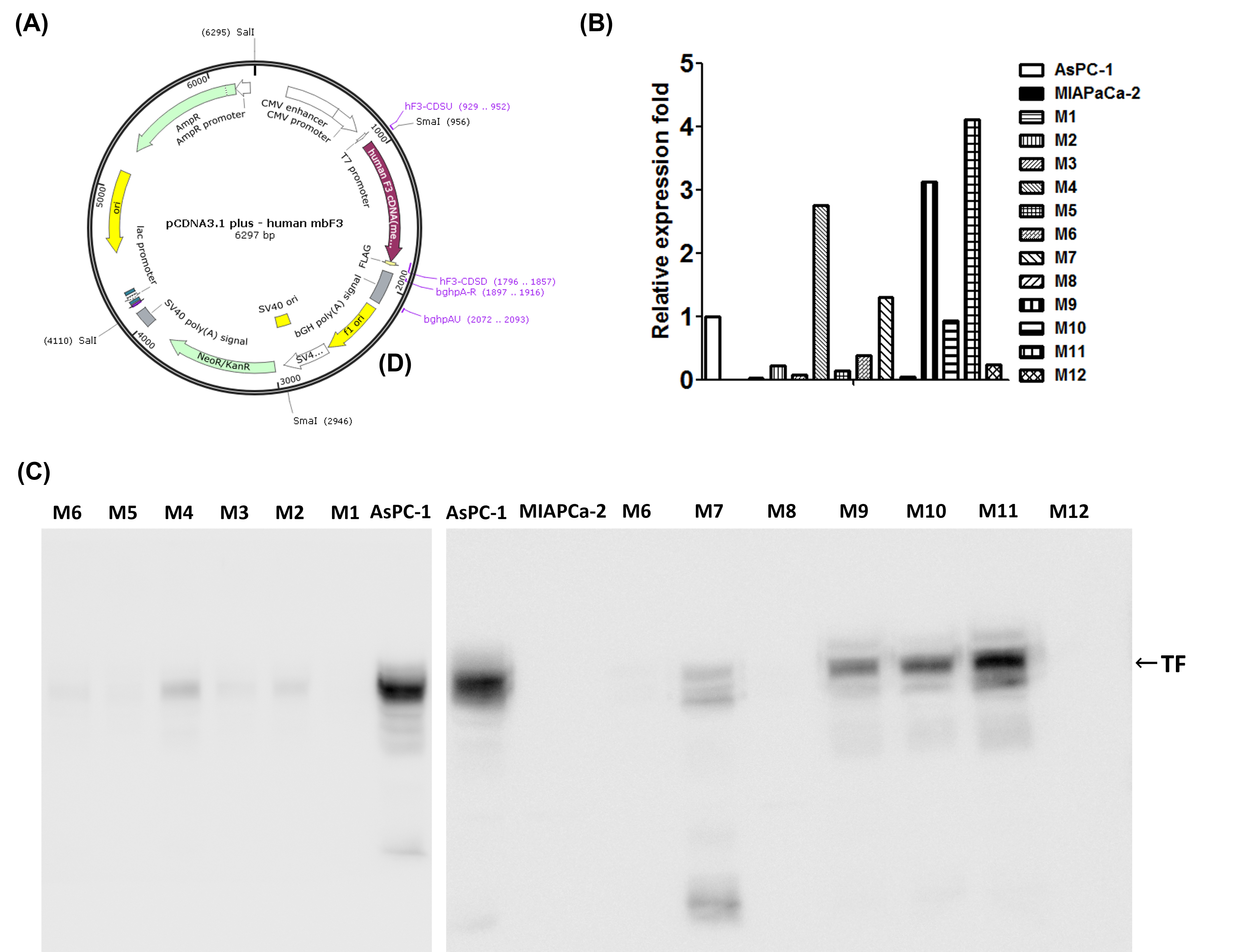


Figure S2: Generate MIA PaCa-2 TF-overexpression cell line (TF^high^). (A) Construction of the TF overexpression plasmid. (B, C) Twelve TF expressing stable clones were isolated by resistance to G418 antibiotics. All clones were analyzed for RNA expression by quantitative RT-PCR(B) and for protein expression by western blotting using human TF antibody (Abcam)(C).


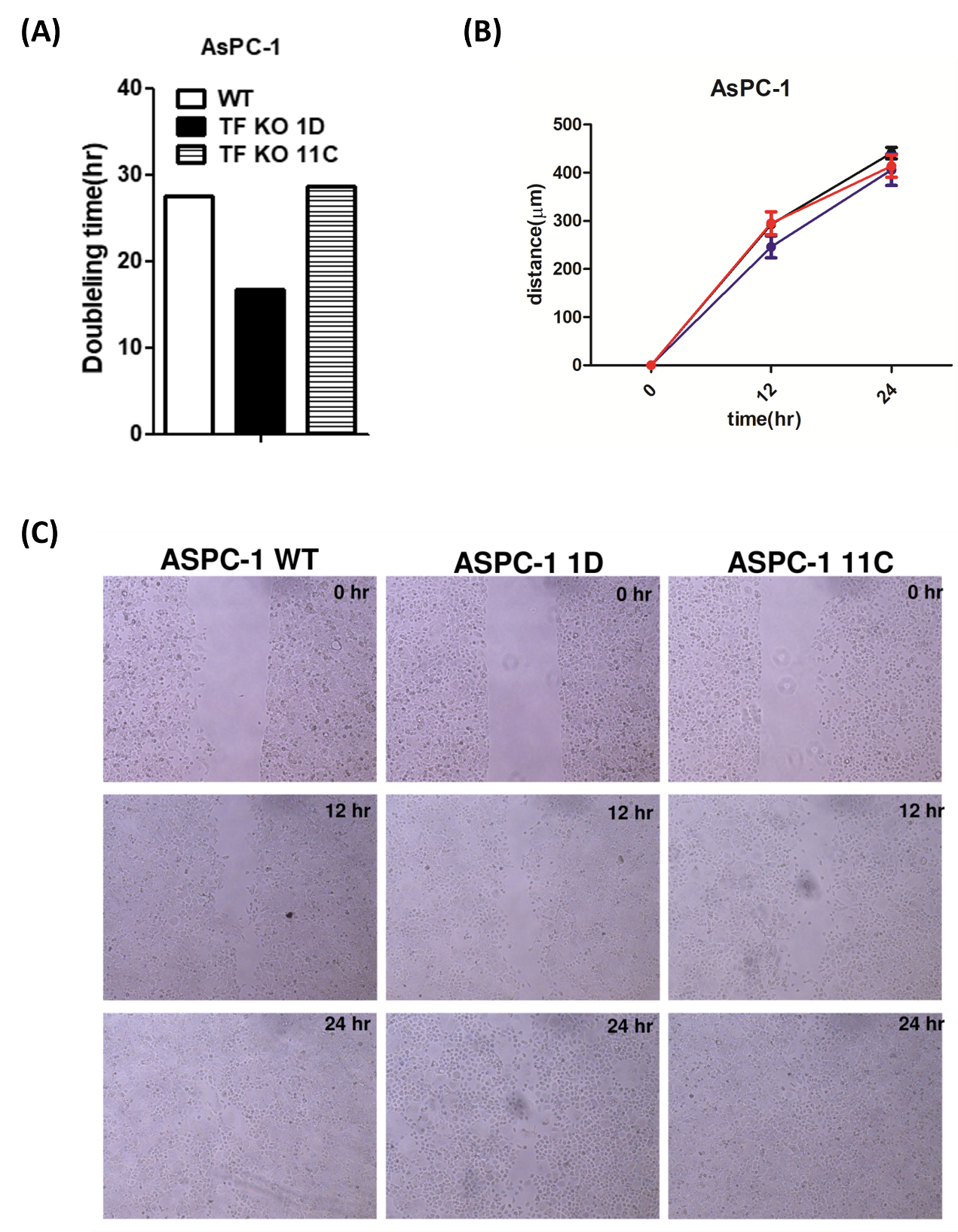


Figure S3: Proliferation and migration of AsPc-1(WT) and AsPC-1(TF^KO^) cell lines. (A) Perform cell viability analysis using WT strain and AsPC-1(TF^KO^) cell lines (1D and 11C) through MTT assay, continuously observing the results for 4 days. (B, C) Migration ability (scratch wound assay). The results were observed at 2, 4, 6, 12 and 24 hours after the cells were cultured. Each time point was repeated in triplicate, quantified in (B), and graphically shown in (C). Transition quantification was performed as described in the literature [1]. Mix clone (1D and 11C) was used in follow-up research.


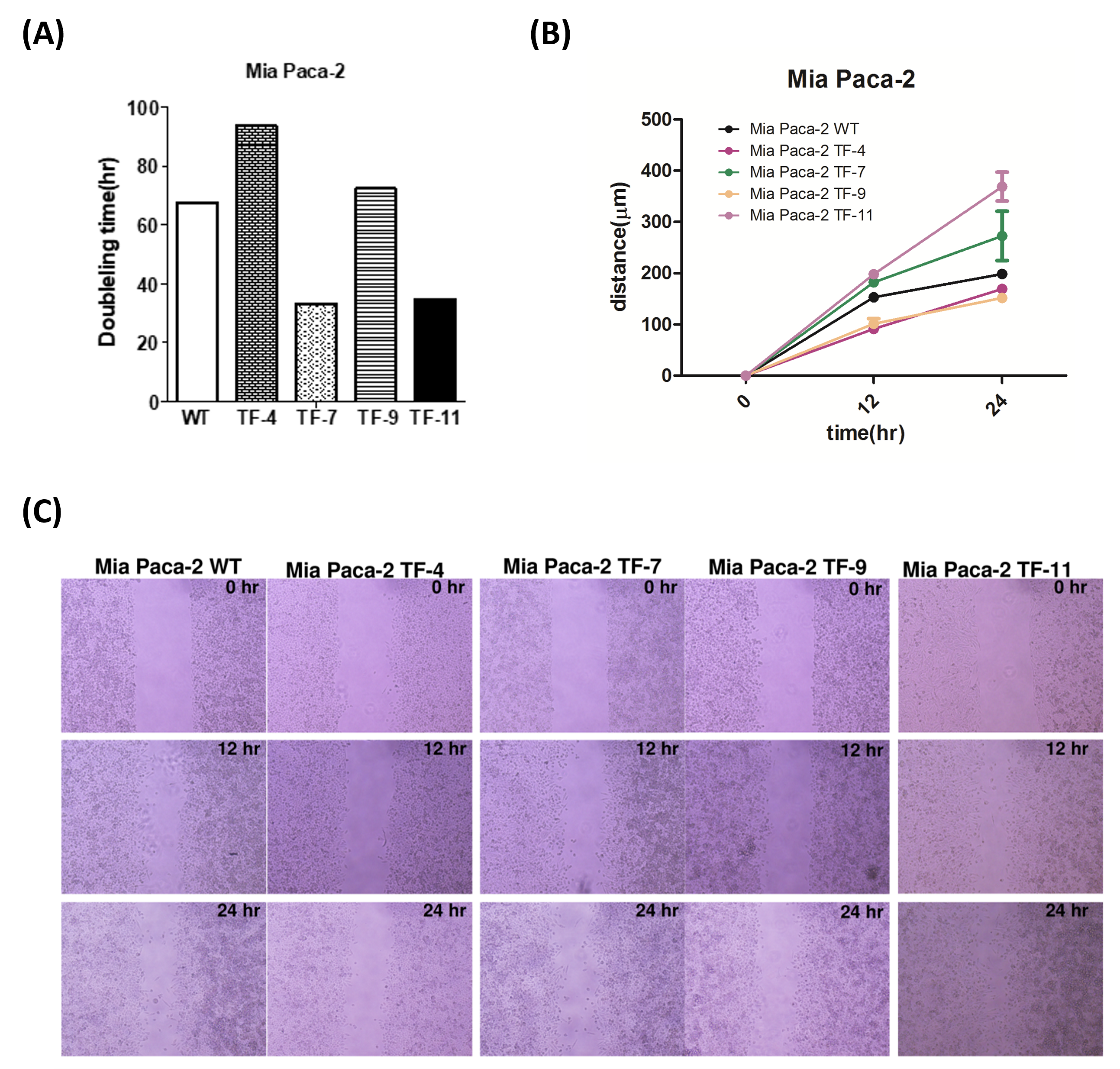


Figure S4: Proliferation and migration of MIA PaCa-2(WT) and MIA PaCa-2(TF^high^) cell lines. (A) Perform cell viability analysis using WT strain and MIA PaCa-2(TF^high^) cell lines (TF-4, 7, 9, 11) through MTT assay, continuously observing the results for 4 days. (B, C) Migration ability (scratch wound assay). The results were observed at 2, 4, 6, 12 and 24 hours after the cells were cultured. Each time point was repeated in triplicate, quantified in (B), and graphically shown in (C). Transition quantification was performed as described in the literature[1]. Clone M11 was used in follow-up research.


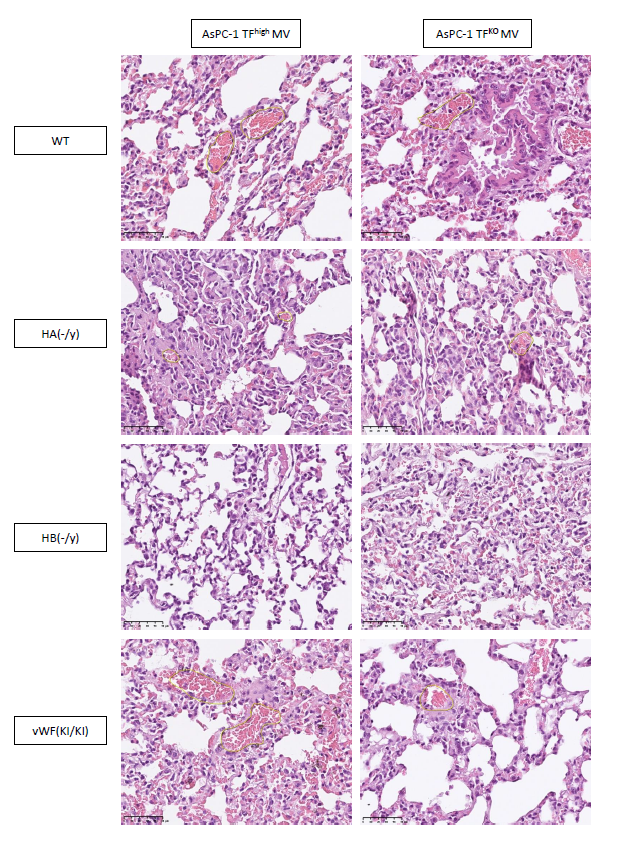


Figure S5: Thrombus formation in lung vessels following the infusion of AsPC-1 TFhigh and TFlow MVs into mice with IVC stenosis.


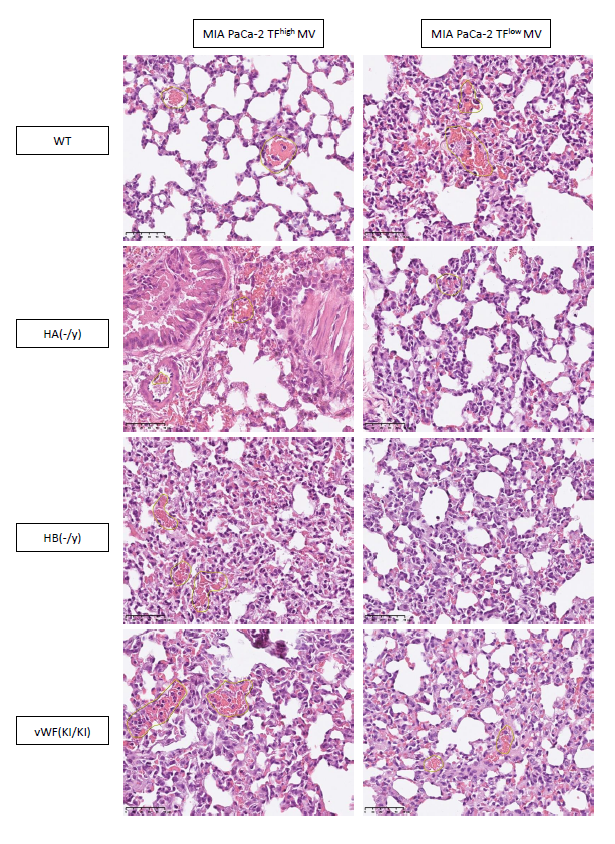


Figure S6: Thrombus within lung vessels after infusion of MIA PaCa-2 TF^high^ and TF^low^ MVs into IVC stenosis mice.

Figure S7:TF supports tumor growth and IVC clots. (A)(B) Representative tumors at day 35 (5-week) in a tumor-bearing mouse model, along with tumor sections stained with H&E stain. Scale bar, 0.5 cm.

Table S1 The number of thrombi within vessels from twenty random observation fields.

**Reference:**

1. Jeong SJ, Kim JH, Lim BJ et al. Inhibition of MUC1 biosynthesis via threonyl-tRNA synthetase suppresses pancreatic cancer cell migration. Experimental &amp; Molecular Medicine 2018; 50: e424-e424. DOI: 10.1038/emm.2017.231
